# Mixed Species Flock, Nest Height, and Elevation Partially Explain Avian Haemoparasite Prevalence in Colombia

**DOI:** 10.1371/journal.pone.0100695

**Published:** 2014-06-20

**Authors:** Angie D. González, Nubia E. Matta, Vincenzo A. Ellis, Eliot T. Miller, Robert E. Ricklefs, H. Rafael Gutiérrez

**Affiliations:** 1 Departamento de Biología, Universidad Nacional de Colombia, Bogotá, Colombia; 2 Department of Biology, University of Missouri-St Louis, St Louis, Missouri, United States of America; Universidade Federal de Minas Gerais, Brazil

## Abstract

The high avian biodiversity present in the Neotropical region offers a great opportunity to explore the ecology of host-parasite relationships. We present a survey of avian haemoparasites in a megadiverse country and explore how parasite prevalences are related to physical and ecological host characteristics. Using light microscopy, we documented the presence of haemoparasites in over 2000 individuals belonging to 246 species of wild birds, from nine localities and several ecosystems of Colombia. We analysed the prevalence of six avian haemoparasite taxa in relation to elevation and the following host traits: nest height, nest type, foraging strata, primary diet, sociality, migratory behaviour, and participation in mixed species flocks. Our analyses indicate significant associations between both mixed species flocks and nest height and *Haemoproteus* and *Leucocytozoon* prevalence. The prevalence of *Leucocytozoon* increased with elevation, whereas the prevalence of *Trypanosoma* and microfilariae decreased. *Plasmodium* and *Haemoproteus* prevalence did not vary significantly with elevation; in fact, both parasites were found up to 3300m above sea level. The distribution of parasite prevalence across the phylogeny of bird species included in this study showed little host phylogenetic signal indicating that infection rates in this system are evolutionarily labile. Vector distribution as well as the biology of transmission and the maintenance of populations of avian haemoparasites deserve more detailed study in this system.

## Introduction

Neotropical countries such as Colombia represent large regions of exceptional biodiversity, with high levels of endemism that are closely related to topographic and climatic complexity [1]. Moreover, Colombia supports the world's highest avian biodiversity [2], offering an ideal opportunity to explore the ecology of host-parasite relationships. Avian haemoparasites are widespread, diverse, and play a central role in avian biology; these haemoparasites influence individual host fitness and reproductive patterns, and can alter bird behaviour [3], [4]. Therefore, understanding factors that drive infection prevalence is an important issue in avian haemoparasite ecology. While some studies have addressed this subject, [5], [6] few have been conducted in Neotropical countries [7]–[9].

Several variables have been shown to correlate with avian haemoparasite transmission, including elevation, latitude, climatic factors, host body size, incubation period, and nest type [6], [8], [10]–[15]. However, when the association between haemoparasite prevalence and host ecological traits (e.g., nest type, diet, and incubation period) is explored, it may be important to account for the potential effects of host phylogenetic relationships because species that share recent ancestry are likely to have similar phenotypes and ecological relationships [16]. Recent increases in the availability of both molecular phylogenies and trait data have stimulated the quantification of phylogenetic signal (“evolutionary conservatism”) in species' traits [17]–[19]. Though most traits exhibit some phylogenetic signal of their shared ancestry, empirical results often indicate that related species resemble each other less, or in some cases more, than expected under an evolutionary model of random Brownian motion [17]. Other models of trait evolution are being developed and tested to account for this non-random evolutionary change (e.g. [20], [21]). How parasite prevalence (proportion of individuals infected in a population) fits into this framework has not been well explored. Those few studies that have empirically addressed the issue have generally found there to be little to no signal [22]–[25], though some of these studies may have suffered from insufficient sample sizes [17], [26], and none have incorporated variance in species' mean prevalence [27]–[29].

The aim of this study was to evaluate the presence of haemoparasites in a Neotropical country and to identify the ecological variables that influence parasite prevalence in this system while analysing the potential effects of host phylogenetic relationships. We show that factors such as elevation, nest height, and participation in mixed species flocks are related to the prevalence of different haemoparasites across hosts in this system.

## Materials and Methods

### Ethical statement

Birds were handled for short periods (less than 10 minutes) to minimize animal stress, and a small amount of blood was obtained. Captured individuals were released immediately after blood sampling. The bird sampling methodology for this study was approved by the Comite de Bioetica de la Facultad de Medicina Veterinaria y Zootécnica of the Universidad Nacional de Colombia (Permit number: CBE-FMVZ-016). Fieldwork was done under permits granted by Unidad Administrativa Especial del Sistema de Parques Nacionales Naturales UAESPNN and Ministerio de Ambiente Vivienda y Desarrollo Territorial.

### Study area, bird sampling, and blood smear analysis

We captured birds with mist-nets in nine distinct areas in Colombia including urban and protected areas with different vegetation types (Table 1). In all, we sampled 2183 individual birds belonging to 41 families, 169 genera and 246 species. Hilty & Brown [30] was used to identify birds in the field and taxonomy follows the South American Classification Committee [31]. Each bird was measured, weighed and blood-sampled from the brachial vein; three thin blood smears were prepared, air dried, then fixed in methanol for 5 minutes and stained with Giemsa (pH 7.2) for 45 minutes. Blood smears were examined first at low magnification (100×) for 10 minutes, and then at high magnification (1000×) for at least 20 minutes. Parasites belonging to the genera *Plasmodium, Haemoproteus, Leucocytozoon, Trypanosoma* and *Hepatozoon* were identified to the genus level; nematode larvae were counted as microfilariae.

**Table 1:** Localities included in this study. Information about vegetation, elevation (m above sea level), and number of birds sampled as well as families and species are given.

Elevation	Coordinates	Sampling date	Season sampled	Vegetation	Sample	Families/genera/species
130	04°31' N 71°31' W	July 1999	Rainy	Ecotone between savannah and gallery forests	102	13/39/46
450	03°21' N 73°56' W	June – July 2000	Rainy	Ecotone between savannah and gallery forests	339	26/55/66
640	04°0.9' N 73°39' W	May – July 1999	Rainy	Tropical rainforest within an urban area	211	19/38/48
2400	04°42' N 75°29' W	June 2011 – January 2012	Dry	High Andean forest	156	15/43/50
2560	04°38' N 74°05' W	September 2009 – October 2012	Dry and rainy*	Vegetation within an urban area	238	15/29/33
2900	04°41' N 73°50' W	February – July 2012	Dry and rainy*	High Andean forest	203	15/32/45
3100	04°37' N 73°43' W	April 2002 – March 2003 December 2008 – October 2009 June – July 2012	Dry and rainy*	Ecotone between high Andean forest and Paramo	340	16/39/48
3300	04°43′ N 75°27′W	April 2011 – January 2012	Dry and rainy*	Ecotone between grass areas and high Andean forest	265	13/33/44
3950	04°46′ N 75°24′W	April 2010 – June 2011	Dry and rainy*	Paramo	325	12/18/19

Asterisk denoted localities where effect of season was evaluated.

### Ecological variables

We obtained the ecological trait data for each avian host from Hilty & Brown [30], Salaman et al. [32] and from field observations. We included the variables and categories as follows (1) (most frequent) nest height: canopy, midstory/understory and ground (2) nest type: open, cavity and closed, (3) main foraging strata: canopy, midstory/understory, understory/ground, ground and air or water, (4) primary diet: frugivore, frugivore/insectivore, insectivore, nectar, nectarivore/insectivore, granivore, omnivore or predator of small vertebrate, (5) sociality: solitary, groups/flocks, (6) participation in mixed species flocks: yes, no, and (7) migratory behaviour: migratory, resident (Table S1). We did not include other variables such as clutch size, incubation period, breeding sociality, breeding season or reproductive status, because either we have no information for the most of the species or the variables showed little variation among species.

### Statistical analysis

#### Localities, habitat sampled and season

Differences in parasite infection between localities, habitat, and season (Table 1) were analysed by Fisher exact tests with simulated p-values (based on 100,000 replicates) when the tables were larger than 2×2, otherwise exact p-values were calculated.

#### Elevation and body mass

We assessed the association between parasite infection and body mass and elevation by logistic regression using each parasite as the response variable and then elevation (m above sea level) or the logarithm (log_10_) of body mass (g) of each individual as predictor variables; host species were included as a covariate in each model.

#### Ecological variables

We were interested in determining the relationship between parasite prevalence, for each parasite genus separately and ecological traits of the avian species in our analysis. We explored the phylogenetic signal of prevalence for each parasite in order to decide if host phylogenetic relationships should be controlled for the ecological trait modelling. To do this, we calculated λ [19] for the prevalence of each parasite taxon in the R package *geiger* [33]. This metric varies from zero (no phylogenetic signal) to one (Brownian motion model of trait evolution) and recently has been shown to be a good descriptor of phylogenetic signal [34]. We used *geiger* to fit two other evolutionary models to our data: a Brownian motion model and a white-noise model. We extracted the log-likelihood values from these models, and compared them to those calculated for the λ and white-noise evolutionary models with a likelihood ratio test. The phylogeny we used in our analysis was a maximum clade credibility tree created from the trees in the posterior distribution of the phylogenetic analysis of Jetz et al. [35] downloaded from www.birdtree.org.

These analyses showed small λ values for all parasite prevalences (Table S2), suggesting low levels of phylogenetic signal in infection rates. Despite the small λ values, *Haemoproteus* and *Plasmodium* exhibited more phylogenetic signal than white-noise models. Other parasite prevalences across the phylogeny could not be distinguished from white-noise models and all parasite prevalences were different than a Brownian motion model of evolution. Due to the low level of phylogenetical signal in parasite prevalence, we modelled prevalence as a function of host ecology without explicitly controlling for host phylogenetic relationships.

We established global models with parasite prevalence as a response variable and all ecological host traits as explanatory variables using generalized linear models with a binomial error structure weighted by species sample size [36]. We used the R package *glmulti* [37] to perform a complete search of all possible models using the ecological trait variables, and we then calculated model-weights based on Akaike's Information Criteria (AIC). Of all possible models we also included a "null" model for comparison, which had no predictor variables [37]. We selected candidate models with delta-AIC scores less than or equal to two, for subsequent model-averaging. From these candidate models we calculated model-averaged parameter estimates, and their associated unconditional variances, standard errors, and 95% confidence intervals following Buckland et al. [38]. We considered predictor variables to be of little importance if the 95% confidence interval of their model-averaged parameter estimate included zero [8].

## Results

**Figure 1:**
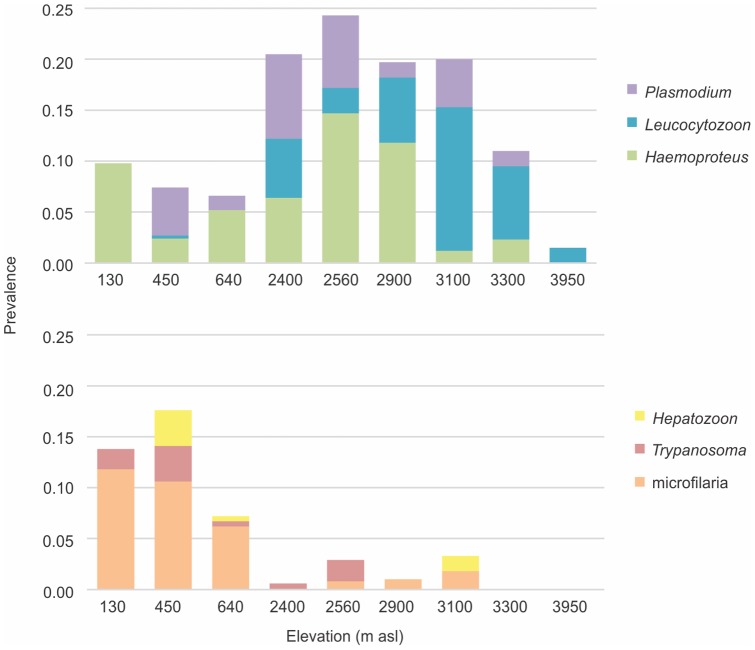
Distribution of the prevalence of six parasite taxa by elevation (m above sea level).

Most of infections recorded here showed a low intensity of infection, which made it difficult to identify all parasites to the species level. The overall haemoparasite prevalence for nine localities in Colombia was 16% (345/2183). Prevalence for each haemoparasite taxon was *Plasmodium* 3% (72/2183), *Haemoproteus* 5% (108/2183), *Leucocytozoon* 5% (101/2183), *Hepatozoon* 1% (18/2183), *Trypanosoma* 1% (21/2183) and microfilariae 3% (71/2183) (Fig. 1). Twelve per cent of the infections were co-infections (41/345). Parasite infection varied significantly across localities and habitat sampled (Fisher's exact test p<0.001 for all parasites for both variables).

Bird families most frequently infected were: Vireonidae 76% (19/25), Turdidae 47% (66/141), Columbidae 32% (12/37), Emberizidae 32% (65/204), Thraupidae 16% (87/552). On the other hand, the well-sampled bird families with lower haemoparasite infection were: Furnariidae 7% (6/82), Hirundinidae 6% (6/98), and Trochilidae 3% (9/289).

### Season, elevation and body mass comparisons

At the site 3100 m above sea level, infection by *Leucocytozoon* was higher in the rainy than in the dry season (Fisher's exact test p  =  0.0178). We did not find other significant differences for any parasite in any of the five localities where two seasons were sampled.

**Figure 2:**
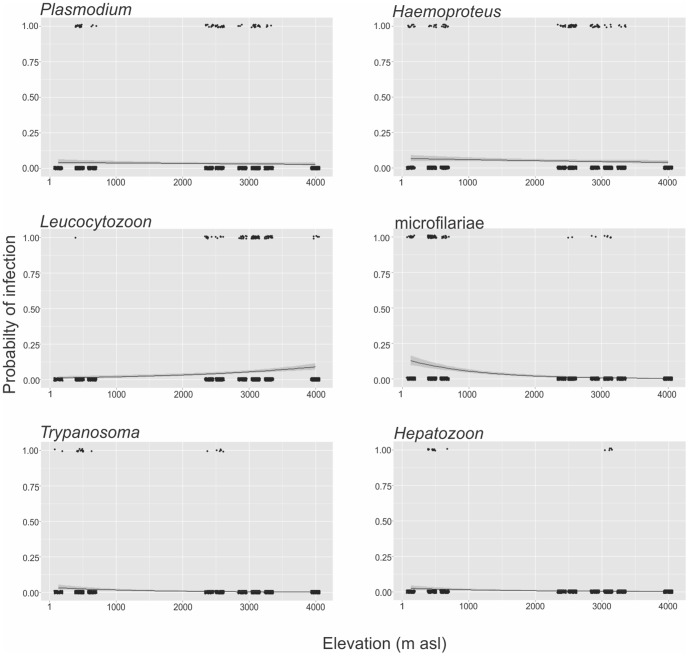
Effect of elevation (m above sea level) on probability of infection for six parasite taxa. Shaded areas about the regression lines indicate 95% confidence intervals. Points were jittered for ease of viewing but may represent only zero or one.

**Figure 3:**
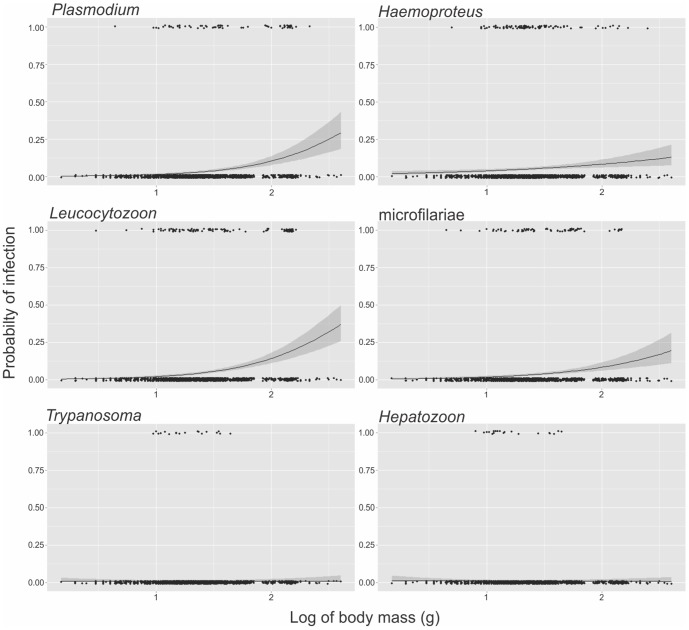
Effect of body mass (log transformed) on probability of infection for six parasite taxa. Shaded areas about the regression lines indicate 95% confidence intervals. Points were jittered for ease of viewing but may represent only zero or one.

We did not find any significant effect of host species on parasite prevalence when the associations between prevalence and elevation and body mass were evaluated and so host species was dropped as a covariate in the final models. *Leucocytozoon* infection was positively associated with elevation, whereas *Hepatozoon*, *Trypanosoma* and microfilariae infection were negatively correlated with elevation (Table 2, Fig. 2). We did not find a significant correlation between parasite prevalence and elevation for *Plasmodium*, *Haemoproteus* or *Hepatozoon* (Table 2, Fig. 2). *Plasmodium*, *Haemoproteus*, *Leucocytozoon* and microfilariae were significantly positively related to host body mass (Table 2, Fig. 3).

**Table 2:** Estimated coefficient of logistic regression between infection of six parasite taxa and elevation and body mass.

	Elevation	Body mass
Parasite	Estimated coefficient	Standard error	p-value	Estimated coefficient	Standard error	p-value
*Plasmodium*	−1.087×10^−4^	8.918×10^−5^	0.223	2.126	0.2773	< 0.0001
*Haemoproteus*	−1.411×10^−4^	7.315×10^−5^	0.0537	0.8131	0.2405	0.0007
*Leucocytozoon*	5.459 ×10^−4^	1.054×10^−4^	< 0.0001	2.0984	0.2394	< 0.0001
microfilariae	−1.07×10^−3^	1.351×10^−4^	< 0.0001	1.6089	0.2779	< 0.0001
*Trypanosoma*	−7.972×10^−4^	2.002×10^−4^	< 0.0001	−0.2626	0.5974	0.66
*Hepatozoon*	−6.634×10^−4^	1.979×10^−4^	0.0008	0.05676	0.62068	0.927

### Life history models

*Plasmodium* was best fitted by a model that included nest type, foraging strata, diet, and sociality as predictor variables (model weight =  0.32, Table 3). However, only diet and nest type showed categories that had confidence intervals that did not include zero. Results showed that species with omnivore diets, and open and closed nest types had higher prevalence of *Plasmodium* (estimated coefficients 1.13, 2.20 and 2.59 respectively, Table S3) than species with other diets and nest types.

**Table 3:** Best fitted models for the relationship between prevalence of six parasite taxa and host ecological variables.

Response Variable	Predictor Variables	Akaike's Information Criteria	Delta Akaike's Information Criteria	Akaike's Information Criteria weights	Cumulative Akaike's Information Criteria weights
*Plasmodium*	NT+FS+D+S	276.67	0.00000	0.32133	0.32133
	NT+FS+D	276.96	0.28594	0.27852	0.59985
	NT+FS+S+D+S+MSF	277.46	0.78276	0.21726	0.81711
	NT+FS+D+MSF	277.80	1.12714	0.18289	1.00000
*Haemoproteus*	NH+NT+D+M+MSF	384.84	0.00000	0.56124	0.56124
	NH+NT+D+S+M+MSF	385.33	0.49239	0.43876	1.00000
*Leucocytozoon*	NT+FS+D+M+MSF	298.47	0.00000	0.66932	0.66932
	NH+NT+FS+D+M+MSF	299.88	1.41026	0.33068	1.00000
*Trypanosoma*	NH+D+S+M+MSF	103.18	0.00000	0.37023	0.37023
	NH+D+S+M	103.54	0.35438	0.31011	0.68034
	NH+D+M	104.79	1.60168	0.16622	0.84656
	NH+D+M+MSF	104.95	1.76160	0.15344	1.00000
microfilariae	D+S+M+MSF	280.73	0.00000	0.62809	0.62809
	D+S+MSF	281.78	1.04808	0.37191	1.00000
*Hepatozoon*	NT+FS+D+S+MSF	88.82	0.00000	0.30592	0.30592
	NH+NT+FS+S+MSF	89.04	0.21585	0.27462	0.58055
	NT+FS+D+S+M+MSF	90.05	1.23016	0.16538	0.74593
	NT+FS+D+MSF	90.45	1.62651	0.13565	0.88158
	NH+NT+FS+D+S+MSF	90.72	1.89818	0.11842	1.00000

Predictor Variables: S: sociality, FS: foraging strata, NT: nest type, MSF: mixed species flocks, D: diet, NH: nest height, M: migratory behaviour. Delta Akaike's Information Criteria equal or lower than 2.0.

Prevalence of *Haemoproteus* was best fitted by a model that included nest height, nest type, diet, migratory status and mixed species flocks as predictor variables (model weight =  0.56). Only the variables diet, mixed species flocks, and nest height showed categories that had confidence intervals that did not include zero. Results showed that species with nest heights of mid-understory and those found in mixed species flocks had higher prevalence of *Haemoproteus* (estimated coefficients 0.74 and 1.23 respectively) than species with other nest heights and those that are not found in mixed species flocks. In addition, we found that bird species with nectarivore-insectivore and mainly insectivore diets (estimated coefficients -2.36 and −0.91 respectively) had lower prevalence than species with other diets.

The best model for the prevalence of *Leucocytozoon* included nest type, foraging strata, diet, migratory status and mixed species flocks (model weight =  0.67). However, only the variables diet, mixed species flocks and migratory status showed categories that had confidence intervals that did not include zero. Results showed that species with resident status, mixed species flocks, insectivore, frugivore-insectivore and omnivore diets had higher prevalence of *Leucocytozoon* (estimated coefficients 1.05, 1.43, 1.19, 1.25 and 1.54 respectively) than species that migrate and have other diets and species that are not found in mixed species flocks.

The prevalence of microfilariae was best fitted by a model that included the predictor variables diet, sociality, migratory status and mixed species flocks (model weight =  0.62). Diet, sociality and mixed species flocks had confidence intervals that did not include zero. Result showed that solitary species and those with diet frugivore-insectivore had higher prevalence of microfilariae (estimated coefficients 0.78 and 0.84 respectively) than species which forms flocks or have other diets. On the other hand, species with nectarivore-insectivore, granivore or insectivore diets and which form mixed species flocks (estimated coefficients −2.47, −1.92, −1.29 and −1.33 respectively) had lower prevalence of microfilariae than species with other diets and those that are not regularly found in mixed species flocks.

*Trypanosoma* was best fitted by a model that included nest height, diet, sociality, migratory status and mixed species flocks as predictor variables (model weight =  0.37). However, only diet and migratory status showed categories that had confidence intervals that did not include zero. Results showed that species with granivore diets and resident status had lower prevalence of *Trypanosoma* (estimated coefficients −2.40 and −1.64 respectively) than species with other diets and those that migrate.

The prevalence of *Hepatozoon* was best fitted by a model that included the predictor variables nest type, foraging strata, diet, sociality and mixed species flocks (model weight  =  0.30), however all of the life history variables included in models had confidence intervals that included zero.

## Discussion

Many vertebrates including birds live in social groups or flocks. One cost of this behaviour can be an increase in the transmission of contact-transmitted pathogens such as H5N1 [39], *Salmonella* [40], mites [41], and vector-transmitted parasites [7]. We found that birds participating in mixed species flocks were more frequently infected with *Haemoproteus* and *Leucocytozoon* than those species that did not. Mixed species flocks are believed to be composed of nuclear species, with facultative follower species. Nuclear species stimulate flock formation and cohesion [42]. In our study, several species including *Anisognathus igniventris*, *Anisognathus somptuosus*, *Atlapetes albinucha*, and *Atlapetes pallidinucha* are all considered nuclear species [43]–[45], and were also frequently infected with *Haemoproteus* and *Leucocytozoon* (Table S1). Furthermore, *Margarornis squamiger* was the only Furnariidae species infected with *Leucocytozoon* and is also a nuclear species [44]. Mixed species flocks are a prevalent characteristic of Andean avian communities [46], and have been hypothesized to help birds obtain more food and avoid predators through group surveillance [47]. However, our data indicate that these benefits may come at a cost of higher rates of parasite infection.

We hypothesized that the prevalence of *Haemoproteus* and *Leucocytozoon* might increase with nest height and foraging stratum since their potential vectors (i.e biting midges and black flies) prefer understory-canopy more than ground strata [48]–[51]. Here we lend support to this hypothesis with our finding that bird species that nest in the mid-understory are more likely to be infected by *Haemoproteus* than species that nest on the ground. This is also in agreement with Fecchio et al. [7] in Brazil. We believe that the general *Culicoides* biting behaviour reported for temperate areas of America [49], [50] may also be exhibited in low latitudinal areas.

We also expected to find higher infection of parasites transmitted by flying, biting dipterans in open-nesting species, since this kind of nest does not provide a physical barrier as much as closed or cavity nests do. On the other hand, parasites such as *Hepatozoon* which are transmitted by non-flying arthrophods [52] could show an association with cavity nests, which are frequently inhabited by mites, ticks and fleas [53], [54]. Nevertheless, we found an increase in *Plasmodium* prevalence in open and closed nesting host species. However, a review of the literature suggests that the association between nest type and haemosporidian prevalence may not be a general one as there are several contrasting findings in Neotropical birds [7], [8].

Concerning migratory behaviour, we expected to find higher prevalence of *Leucocytozoon* in migratory birds for two reasons: first *Leucocytozoon* prevalence in the Neartic region is higher than that reported for the Neotropical region [55], [56]; and second, the energetic cost of migration could result in immune suppression [57] and could potentially result in an increase in parasite intensity [58]. Our results show that resident birds are more infected than migratory birds, but these results should be interpreted cautiously due to the low number of migratory birds in our sample (5%).

Here we found a significant association between *Leucocytozoon* and the rainy season in one locality, and found no other parasite prevalence-season associations. A study made in Brazil [59] reported more *Haemoproteus* infections in the rainy season compared with the dry season. Parasite prevalence may respond to more fine scale environmental cues than the categories of season can afford; future studies should consider quantitatively measuring temperature, rainfall and relative humidity during sampling dates.

We expected a negative relationship between parasite prevalence and elevation based on previous studies [4], [10], [60], with the exception of *Leucocytozoon* which has been documented to increase in prevalence with increasing elevation [61]. Nevertheless, we found a different pattern of distribution for each parasite taxon with respect to elevation. *Leucocytozoon* prevalence, as expected, showed a positive association with elevation, varying from absence in lowlands (in resident birds), to its highest prevalence at 3100 m above sea level; *Leucocytozoon* was even found as high as 3950 m above sea level (Fig. 1). Van Rooyen et al. [61] also found a positive association in Switzerland between *Leucocytozoon* and elevation. We also demonstrated the presence of *Plasmodium* and *Haemoproteus* up to 3300 m above sea level, and found no significant relationship with elevation. This is in contrast to previous studies that often report a negative relationship between the prevalence of *Plasmodium* and *Haemoproteus* and elevation [10], [15], [60]. The patterns of haemoparasite distribution with elevation, and all the changes in vegetation and fauna that this represents in Neotropical countries, will likely be elucidated by more studies on the biology and distribution of hematophagous fauna and vector identification across different ecosystems.

The overall prevalence in our system was low when compared with similar parasite surveys in the Nearctic region, in keeping with previous results documenting low avian haemoparasite prevalence in Neotropical countries based on screening blood smears [9], [56], [59], [62]–[78] (Table S4). We assume that if we had used PCR, parasite prevalence would have been higher, however, microscopy allowed identification of several taxonomic groups at the same time and avoided including abortive infections and non-real host-parasite associations that are not distinguished by PCR [79], [80].

Hypotheses to explain the low parasite prevalence in Neotropical countries compared to temperate regions include: (1) the great avian biodiversity present in the Neotropical region may lead to a dilution effect [81], decreasing the density of susceptible hosts. Such an effect would provide less opportunity for transmission [82]; (2) the life-history strategies of tropical birds, which are typically longer-lived than species at high latitudes, might include stronger immune defences against infection [14].

As in previous empirical studies, we found little phylogenetic signal in species' prevalences for the six parasites examined. Two of six calculated λ values were greater than those expected given a white-noise evolutionary model (Table S2), suggesting a small but significant degree of phylogenetic signal in parasite prevalence, however, these results should be interpreted cautiously. We suggest that: (1) more work is needed to address calculations of phylogenetic signal with means and variances in proportional traits, and (2) phylogenetic signal may be reduced by a variety of factors including high rates of host-switching in blood parasites, divergent selection in sympatry, and migratory behaviour [83], [84].

In conclusion, our study presents a survey of avian haemoparasites in a megadiverse region, and explores how ecological variables are related to parasite prevalences in wild birds. Our results indicate significant associations between *Leucocytozoon* and *Haemoproteus* prevalence and mixed species flocks, as well as a significant association between *Haemoproteus* prevalence and nest height. *Plasmodium* and *Haemoproteus* prevalences did not decrease with increasing elevation as expected from previous studies, whilst *Leucocytozoon* is strictly distributed at highlands for resident bird species. Parasite prevalence showed very little phylogenetic signal throughout the host phylogeny as expected from previous studies. Despite the growing information on avian haemoparasites, we are still far from identifying which factors drive their transmission; most appear to be idiosyncratically related to prevalence across the landscape. Nevertheless, here we identify biotic and abiotic factors related to haemoparasite prevalence in Neotropical ecosystems.

## Supporting Information

Table S1 **Database used in this study, abiotic variables as well as host ecological variables obtained from Hilty & Brown** [30] **, Salaman et al.** [32] **and field observation are provided.** Bird family and species. Infection with six parasite taxa: infected (1), non-infected (0). Elevation (m above sea level). Season sampled: rainy, dry. Habitat sampled: Ecotone between savannah and gallery forests (E_S_GF), Tropical rainforest within an urban area (TRF_U), Vegetation within an urban area (V_U), High Andean forest (AF), Ecotone between grass areas and high Andean forest (E_G_AF), Ecotone between high Andean forest and Paramo (E_AF_P), Paramo (P). Body mass (g). Nest height: canopy, midstory/understory, ground. Nest type: open, cavity, closed. Foraging strata: canopy, midstory/understory, understory/ground, ground, air or water. Diet: frugivore, frugivore/insectivore, insectivore, nectar, nectarivore/insectivore, granivore, omnivore or predator of small vertebrate. Sociality: solitary, groups/flocks. Mixed species flocks: no (0), yes (1). Migratory behaviour: migrant, resident.
(XLSX)

Table S2 **The calculated lambda values for the prevalence of each parasite and the P-values from likelihood ratio tests comparing the fit of lambda to that of the white-noise and Brownian motion models of evolution.**
(DOCX)

Table S3 **Model-averaged parameter estimates for each of the parasite prevalence in relation to the life history traits (see Materials and Methods).** Asterisk denoted variable with confidence intervals that did not include zero.
(DOCX)

Table S4 **Comparison of parasite prevalence of this study and other developed in the Neotropical region.** Others include *Hepatozoon*, *Atoxoplasma*, *Lankesterella* and unidentified parasites.
(DOCX)
