# Supplementary material for: Mixed Species Flock, Nest Height, and Elevation Partially Explain Avian Haemoparasite Prevalence in Colombia
Source: PLoS One. 2014 Jun 20;9(6):e100695. doi: 10.1371/journal.pone.0100695 (PMC4065061; doi:10.1371/journal.pone.0100695)
Supplement: Table S2 — The calculated lambda values for the prevalence of each parasite and the P-values from likelihood ratio tests comparing the fit of lambda to that of the white-noise and Brownian motion models of evolution. (DOCX) [file pone.0100695.s002.docx]

**Table S2. The calculated lambda values for the prevalence of each parasite and the P-values from likelihood ratio tests comparing the fit of lambda to that of the white-noise and Brownian motion models of evolution.**

| infection | Observed Lambda | P-value  White-noise | P-value  Brownian motion |
| --- | --- | --- | --- |
| *Plasmodium* | 0.4494 | 0.0024 | < 0.00001 |
| *Leucocytozoon* | < 0.0001 | 1 | < 0.00001 |
| *Haemoproteus* | 0.5082 | < 0.00001 | < 0.00001 |
| *Trypanosoma* | 0.0664 | 0.1322 | < 0.00001 |
| microfilariae | < 0.0001 | 1 | < 0.00001 |
| *Hepatozoon* | <0.0001 | 0.9991 | < 0.00001 |
