# Supplementary material for: Mixed Species Flock, Nest Height, and Elevation Partially Explain Avian Haemoparasite Prevalence in Colombia
Source: PLoS One. 2014 Jun 20;9(6):e100695. doi: 10.1371/journal.pone.0100695 (PMC4065061; doi:10.1371/journal.pone.0100695)
Supplement: Table S3 — Model-averaged parameter estimates for each of the parasite prevalence in relation to the life history traits (see Materials and Methods). Asterisk denoted variable with confidence intervals that did not include zero. (DOCX) [file pone.0100695.s003.docx]

**Table S3. Model-averaged parameter estimates for each of the parasite prevalence in relation to the life history traits (see Materials and Methods).** Asterisk denoted variable with confidence intervals that did not include zero.

| **Response Variable** | **Predictor Variable** | **Model-averaged Estimate** | **Unconditional Variance** | **Standard Error** | **Unconditional 95% CI (+/-)** | **Unconditional Lower 95% CI** | **Unconditional Upper 95% CI** |
| --- | --- | --- | --- | --- | --- | --- | --- |
| *Plasmodium* | Diet: omnivore | 1.13* | 0.29 | 0.54 | 1.07 | 0.06 | 2.19 |
| *Plasmodium* | Nest type: open | 2.20* | 1.17 | 1.08 | 2.13 | 0.07 | 4.34 |
| *Plasmodium* | Nest type: closed | 2.59* | 1.23 | 1.11 | 2.18 | 0.41 | 4.78 |
| *Plasmodium* | Intercept | -6.24 | 1.85 | 1.36 | 2.68 | -8.92 | -3.56 |
| *Plasmodium* | Diet: granivore | -1.10 | 0.41 | 0.64 | 1.26 | -2.36 | 0.16 |
| *Plasmodium* | Mixed flocks | 0.17 | 0.07 | 0.27 | 0.53 | -0.37 | 0.70 |
| *Plasmodium* | Sociality: solitary | 0.29 | 0.14 | 0.37 | 0.73 | -0.43 | 1.02 |
| *Plasmodium* | Diet: insectivore | 0.05 | 0.29 | 0.54 | 1.07 | -1.01 | 1.12 |
| *Plasmodium* | Diet: nectarivore-insectivore | 0.03 | 0.41 | 0.64 | 1.26 | -1.23 | 1.29 |
| *Plasmodium* | Diet: frugivore-insectivore | 0.56 | 0.28 | 0.53 | 1.04 | -0.49 | 1.60 |
| *Plasmodium* | Foraging strata: mid-understory | -0.52 | 1.29 | 1.14 | 2.24 | -2.76 | 1.72 |
| *Plasmodium* | Foraging strata: canopy | 0.91 | 1.32 | 1.15 | 2.27 | -1.36 | 3.18 |
| *Plasmodium* | Foraging strata: understory-ground | 1.04 | 1.25 | 1.12 | 2.20 | -1.16 | 3.24 |
| *Plasmodium* | Foraging strata: ground | 1.23 | 1.29 | 1.14 | 2.24 | -1.01 | 3.46 |
| *Haemoproteus* | Diet: nectarivore-insectivore | -2.36* | 0.26 | 0.51 | 1.00 | -3.35 | -1.36 |
| *Haemoproteus* | Diet: insectivore | -0.91* | 0.14 | 0.38 | 0.75 | -1.66 | -0.16 |
| *Haemoproteus* | Mixed flocks | 0.74* | 0.08 | 0.28 | 0.56 | 0.18 | 1.30 |
| *Haemoproteus* | Nest height: mid-understory | 1.23* | 0.11 | 0.33 | 0.66 | 0.57 | 1.88 |
| *Haemoproteus* | Migrant: resident | -0.90* | 0.12 | 0.35 | 0.69 | -1.60 | -0.21 |
| *Haemoproteus* | Diet: omnivore | -0.82 | 0.24 | 0.49 | 0.96 | -1.78 | 0.14 |
| *Haemoproteus* | Diet: frugivore-insectivore | -0.56 | 0.14 | 0.38 | 0.75 | -1.31 | 0.19 |
| *Haemoproteus* | Diet: granivore | -0.28 | 0.16 | 0.41 | 0.80 | -1.08 | 0.52 |
| *Haemoproteus* | Sociality: solitary | 0.14 | 0.04 | 0.21 | 0.41 | -0.27 | 0.54 |
| *Haemoproteus* | Nest height: ground | -0.30 | 0.44 | 0.66 | 1.30 | -1.60 | 1.00 |
| *Haemoproteus* | Intercept | -17.91 | 647994 | 805 | 1586 | -1604 | 1568 |
| *Haemoproteus* | Nest type: closed | 12.43 | 647994 | 805 | 1586 | -1574 | 1598 |
| *Haemoproteus* | Nest type: open | 15.41 | 647993 | 805 | 1586 | -1571 | 1601 |
| *Leucocytozoon* | Migrant: resident | 1.05* | 0.23 | 0.48 | 0.94 | 0.11 | 1.99 |
| *Leucocytozoon* | Mixed flocks | 1.34* | 0.11 | 0.34 | 0.66 | 0.68 | 2.01 |
| *Leucocytozoon* | Diet: insectivore | 1.19* | 0.18 | 0.42 | 0.83 | 0.35 | 2.02 |
| *Leucocytozoon* | Diet: frugivore-insectivore | 1.25* | 0.19 | 0.43 | 0.85 | 0.40 | 2.11 |
| *Leucocytozoon* | Diet: omnivore | 1.54* | 0.21 | 0.46 | 0.90 | 0.65 | 2.44 |
| *Leucocytozoon* | Diet: granivore | -1.11 | 0.38 | 0.61 | 1.21 | -2.31 | 0.10 |
| *Leucocytozoon* | Nest height: mid-understory | -0.05 | 0.02 | 0.14 | 0.28 | -0.33 | 0.23 |
| *Leucocytozoon* | Diet: nectarivore-insectivore | -0.52 | 0.35 | 0.59 | 1.17 | -1.68 | 0.65 |
| *Leucocytozoon* | Nest height: ground | -0.32 | 0.26 | 0.51 | 1.00 | -1.32 | 0.68 |
| *Leucocytozoon* | Nest type: closed | 15.13 | 1413241 | 1189 | 2342 | -2327 | 2357 |
| *Leucocytozoon* | Nest type: open | 17.85 | 1413240 | 1189 | 2342 | -2324 | 2360 |
| *Leucocytozoon* | Foraging strata: mid-understory | 15.20 | 2414964 | 1554 | 3062 | -3047 | 3077 |
| *Leucocytozoon* | Foraging strata: ground | 15.89 | 2414964 | 1554 | 3062 | -3046 | 3078 |
| *Leucocytozoon* | Foraging strata: canopy | 16.25 | 2414964 | 1554 | 3062 | -3046 | 3078 |
| *Leucocytozoon* | Foraging strata: understory-ground | 17.02 | 2414964 | 1554 | 3062 | -3045 | 3079 |
| *Leucocytozoon* | Intercept | -38.55 | 3828205 | 1957 | 3855 | -3894 | 3816 |
| *Trypanosoma* | Migrant: resident | -1.64* | 0.37 | 0.61 | 1.20 | -2.83 | -0.44 |
| *Trypanosoma* | Diet: granivore | -2.40* | 1.40 | 1.18 | 2.33 | -4.73 | -0.06 |
| *Trypanosoma* | Intercept | -2.84 | 1.36 | 1.17 | 2.30 | -5.14 | -0.55 |
| *Trypanosoma* | Mixed flocks | -0.42 | 0.30 | 0.54 | 1.07 | -1.49 | 0.65 |
| *Trypanosoma* | Diet: frugivore-insectivore | -0.74 | 0.59 | 0.77 | 1.52 | -2.25 | 0.78 |
| *Trypanosoma* | Diet: insectivore | -0.42 | 0.46 | 0.68 | 1.33 | -1.75 | 0.91 |
| *Trypanosoma* | Nest height: mid-understory | 0.36 | 0.29 | 0.54 | 1.06 | -0.69 | 1.42 |
| *Trypanosoma* | Sociality: solitary | 0.90 | 0.81 | 0.90 | 1.77 | -0.86 | 2.67 |
| *Trypanosoma* | Nest height: ground | -18.90 | 11999410 | 3464 | 6824 | -6843 | 6805 |
| *Trypanosoma* | Diet: nectarivore-insectivore | -20.32 | 20377570 | 4514 | 8893 | -8914 | 8873 |
| *Trypanosoma* | Diet: omnivore | -19.65 | 23530210 | 4851 | 9556 | -9576 | 9537 |
| *Microfilarie* | Diet: nectarivore-insectivore | -2.47* | 0.45 | 0.67 | 1.33 | -3.80 | -1.15 |
| *Microfilarie* | Diet: granivore | -1.92* | 0.31 | 0.56 | 1.10 | -3.02 | -0.82 |
| *Microfilarie* | Mixed flocks | -1.20* | 0.10 | 0.32 | 0.63 | -1.83 | -0.56 |
| *Microfilarie* | Diet: insectivore | -1.33* | 0.18 | 0.43 | 0.84 | -2.17 | -0.49 |
| *Microfilarie* | Sociality: solitary | 0.78* | 0.10 | 0.32 | 0.64 | 0.14 | 1.42 |
| *Microfilarie* | Diet: frugivore-insectivore | 0.84* | 0.16 | 0.40 | 0.78 | 0.06 | 1.62 |
| *Microfilarie* | Intercept | -3.51 | 0.69 | 0.83 | 1.63 | -5.14 | -1.88 |
| *Microfilarie* | Diet: omnivore | -0.14 | 0.20 | 0.45 | 0.88 | -1.02 | 0.75 |
| *Microfilarie* | Migrant: resident | 0.63 | 0.49 | 0.70 | 1.37 | -0.74 | 2.01 |
| *Hepatozoon* | Nest height: mid-understory | -0.77 | 1.27 | 1.13 | 2.22 | -2.98 | 1.45 |
| *Hepatozoon* | Foraging strata: understory-ground | 0.22 | 2.26 | 1.50 | 2.96 | -2.75 | 3.18 |
| *Hepatozoon* | Sociality: solitary | -4.33 | 182091 | 427 | 841 | -845 | 836 |
| *Hepatozoon* | Foraging strata: mid-understory | -5.54 | 182900 | 428 | 843 | -848 | 837 |
| *Hepatozoon* | Diet: insectivore | -5.49 | 182925 | 428 | 843 | -848 | 837 |
| *Hepatozoon* | Diet: frugivore-insectivore | -3.53 | 182145 | 427 | 841 | -844 | 837 |
| *Hepatozoon* | Mixed flocks | 5.96 | 182126 | 427 | 841 | -835 | 847 |
| *Hepatozoon* | Migrant: resident | 2.95 | 946678 | 973 | 1917 | -1914 | 1920 |
| *Hepatozoon* | Nest type: open | -12.31 | 3919445 | 1980 | 3901 | -3913 | 3888 |
| *Hepatozoon* | Nest type: closed | -11.01 | 3919322 | 1980 | 3900 | -3912 | 3889 |
| *Hepatozoon* | Nest height: ground | -9.50 | 3920598 | 1980 | 3901 | -3911 | 3892 |
| *Hepatozoon* | Diet: nectarivore-insectivore | -15.77 | 13501710 | 3674 | 7239 | -7255 | 7224 |
| *Hepatozoon* | Diet: omnivore | -17.44 | 18694350 | 4324 | 8519 | -8536 | 8501 |
| *Hepatozoon* | Diet: granivore | -18.36 | 20710700 | 4551 | 8966 | -8985 | 8948 |
| *Hepatozoon* | Foraging strata: ground | -18.28 | 23193910 | 4816 | 9489 | -9507 | 9470 |
| *Hepatozoon* | Foraging strata: canopy | -31.84 | 35997130 | 6000 | 11821 | -11853 | 11789 |
