# Supplementary material for: Mixed Species Flock, Nest Height, and Elevation Partially Explain Avian Haemoparasite Prevalence in Colombia
Source: PLoS One. 2014 Jun 20;9(6):e100695. doi: 10.1371/journal.pone.0100695 (PMC4065061; doi:10.1371/journal.pone.0100695)
Supplement: Table S4 — Comparison of parasite prevalence of this study and other developed in the Neotropical region. Others include Hepatozoon, Atoxoplasma, Lankesterella and unidentified parasites. (DOCX) [file pone.0100695.s004.docx]

**Table S4. Comparison of parasite prevalence of this study and other developed in the Neotropical region.** Others include *Hepatozoon*, *Atoxoplasma*, *Lankesterella* and unidentified parasites.

| Geographic region | Number studied | Number infected (prevalence) | *Plasmodium* | *Haemoproteus* | *Leucocytozoon* | microfilariae | *Trypanosome* | Others | Reference |
| --- | --- | --- | --- | --- | --- | --- | --- | --- | --- |
| Colombia | 2183 | 345 (16%) | 72 (3%) | 108 (5%) | 101 (5%) | 71 (3%) | 21 (1%) | 18 (1%) | This study |
| Colombia | 421 | 30 (7.1%) | 3 (0.7%) | 13 (3.1%) | 3 (0.7%) | 10 (2.3%) | 0 | 3 (0.7%) | [63] |
| Colombia | 324 | 82 (24%) | 36 (10.5%) | 11 (3.24%) | 1 (0.3%) | 36 (10.5%) | 12 (3.5%) | 18 (5.3%) | [61] |
| Colombia | 302 | 28 (9.3%) | 17 (5.6%) | 8 (2.6%) | 1 (0.3%) | 3 (1%) | 0 | 0 | [69] |
| Colombia | 64 | 5 (8%) | 2 (2%) | 2 (3%) | 1 (2%) | 2 (3%) | 0 | 0 | [74] |
| Venezuela | 8565 | 285 (3.3%) | 60 (0.7%) | 214 (2.5%) | 0 | 7 (0.08%) | 4 (0.05%) | 0 | [68] |
| Venezuela | 3944 | 178 (4.5%) | 26 (0.6%) | 135 (3.4%) | 1 (0.02%) | 3 (0.08%) | 5 (0.13%) | 8 (0.2%) | [67] |
| Panamá | 3715 | 661  (17.8%) | 197  (5.3%) | 334  (9.0%) | 19  (0.5%) | 108  (2.9%) | 74  (2%) | 7  (0.2%) | [73] |
| Brazil | 15574 | 1240 (8.0%) | 101 (0.4%) | 523 (3,3%) | 10 (0.06%) | 412 (2.6%) | 184 (1.2%) | 103 (0.6%) | [76] |
| Brazil | 3449 | 268 (7.8%) | 40 (1.6%) | 120 (3.5%) | 0 | 89 (2.5%) | 29 (0.8%) | 6 (0.2%) | [64] |
| Brazil | 925 | 146 (15.8%) | 80 (8.7%) | 31 (3.4%) | 0 | 3 (0.3%) | 34 (3.7%) | 0 | [58] |
| Brazil | 508 | 35 (6.9%) | 8.1 (1.6%) | 27 (5.3%) | 0 | 0 | 0 | 0 | [66] |
| Brazil | 423 | 23 (5.4%) | 0 | 0 | 0 | 23 (5.4%) | 0 | 0 | [70] |
| Brazil | 166 | 11 (6.6%) | 0 | 0 | 0 | 11 (6.6%) | 0 | 0 | [72] |
| Costa Rica | 479 | 51 (10.6%) | 2 (0.4%) | 45 (9.4%) | 2 (0.4%) | 5 (1%) | 1 (0.2%) | 0 | [77] |
| Costa Rica | 354 | 44 (12.4%) | 2 (0.6%) | 17 (4.8%) | 1 (0.3%) | 27 (7.6%) | 7 (2.0%) | 0 | [75] |
| Costa Rica | 248 | 22 (8.8%) | 0 | 2 (0.8%) | 0 | 0 | 20 (8.1%) | 0 | [62] |
| Bolivia | 641 | 33 (5.1%) | 7 (1.9%) | 9 (1.4%) | 0 | 12 (1.9%) | 2 (0.3%) | 4 (0.3%) | [65] |
| Neotropical region review | 14396 | 2482 (17.2%) | 609  (4.2%) | 1457  (10.1%) | 54  (0.4%) | 440  (3.1%) | 196  (1.4%) | 73 (0.5%) | [55] |
